# Protocol for developing the nutrition dataset for the international spinal cord society: an international eDelphi approach

**DOI:** 10.1038/s41393-025-01102-z

**Published:** 2025-07-04

**Authors:** Priya Iyer, Gary J. Farkas, Sherri L. LaVela, Shashivadan P. Hirani, Samford Wong

**Affiliations:** 1 Nutrition and Dietetics, Susan Wakil School of Nursing and Midwifery, Faculty of Medicine and Health, University of Sydney, Sydney, NSW Australia; 2 Charles Perkins Centre, University of Sydney, Sydney, NSW Australia; 3 Department of Physical Medicine and Rehabilitation, University of Miami Miller School of Medicine, Miami, FL USA; 4 Miami Project to Cure Paralysis, University of Miami Miller School of Medicine, Miami, FL USA; 5 Center of Innovation for Complex Chronic Healthcare (CINCCH), Department of Veterans Affairs, Edward Hines Jr. VA Hospital, Hines, IL USA; 6 Department of Physical Medicine and Rehabilitation, Feinberg School of Medicine, Northwestern University, Chicago, IL USA; 7 School of Health & Medical Sciences, City & St George’s, University of London, London, UK; 8 National Spinal Injuries Centre, Stoke Mandeville Hospital, Aylesbury, UK; 9 Royal Buckinghamshire Hospital, Aylesbury, UK

**Keywords:** Spinal cord diseases, Nutrition

## Abstract

**Study design:**

An eDelphi survey.

**Objective:**

To develop the Spinal Cord Injury (SCI) basic and extended nutrition datasets for adults with SCI for the International Spinal Cord Society (ISCoS).

**Setting:**

This international eDelphi study, administered in Australia, will be conducted virtually, overseen by a Research Advisory Group.

**Methods:**

An expert panel will be recruited internationally to participate in a three-round eDelphi survey to develop the ISCoS basic and extended nutrition datasets. An a priori criterion will be implemented, defining strong consensus as an interquartile range (IQR) ≤ 1 and consensus as an IQR > 1/≤2. Mean and standard deviation will be calculated to measure convergence and stability depending on the data. Agreement will be determined as ≥ 80% per statement (Likert scale ratings of 4 and 5). A content analysis approach will be utilised to synthesise free-text responses.

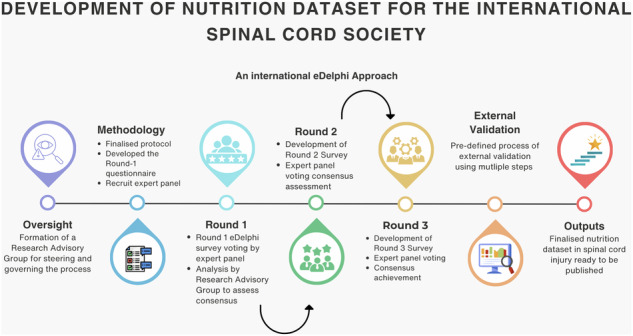

## Introduction

A spinal cord injury (SCI) resulting from trauma or disease disrupts spinal cord pathways, leading to paralysis and adverse alterations in whole-body metabolism. The rapid onset of immobility and dysfunctional physiological health stemming from autonomic dysfunction [1], sublesional myopenia [2], imposed or adopted sedentary behaviour [3], and the overconsumption of energy [4] relative to reduced total energy expenditure [5, 6] contributes to the development of neurogenic obesity [7, 8]. These aberrant injury-related changes heighten cardiometabolic risk [8, 9]. Additionally, the prevalence of malnutrition in this population ranges from 40% to over 60% across studies, largely due to considerable variation in the methods used for assessment and diagnosis of malnutrition [10–12].

The wide variability of nutrition-related data stems from a lack of standardisation in study procedures, including the assessment methods, tools, and instruments used to evaluate nutritional outcomes. A standardised nutritional dataset would allow for the evaluation of outcomes, facilitate comparisons across studies and sub-populations, enable the incorporation of such a tool into clinical settings, and support progression in the field of SCI nutrition care. While the International Spinal Cord Society (ISCoS) has developed common datasets for standardised reporting in SCI related to cardiovascular and endocrine and metabolism health [13], these datasets do not capture diet-related and nutritional health status assessments and outcomes.

The objective of the proposed study therefore is to develop both a basic and extended nutrition dataset for adults with SCI through an electronic Delphi (eDelphi) approach. This will involve a diverse panel of experts’ including clinicians and researchers with extensive experience in nutrition for SCI. For this study, the basic dataset is defined as those items considered essential to be collected at a minimum. Whereas the extended dataset will include items which could be beneficial to collect but not critical for minimum standardised care. We hypothesise that an international panel of experts in SCI and nutrition will reach consensus on a set of specific, clinically relevant items to be included in both basic and extended nutrition datasets for adults with SCI. Furthermore, these datasets will encompass key nutrition areas such as anthropometric measurements, dietary intake assessment, and nutrition-related complications specific to SCI. The basic dataset will offer consistent data suited for standard clinical practice, whereas the extended dataset will provide more detailed and specific information, enhancing the depth and applicability for research outputs. The eDelphi approach is well-suited for creating a comprehensive SCI nutrition dataset as expert-derived information tends to be practical, enabling consensus through expert opinion and ensuring usability that reflects real-world applications [14]. The finalised nutrition dataset would be utilised alongside other ISCoS datasets, such as the core [15], endocrine and metabolic [16] datasets or any other relevant datasets as appropriate.

## Methods

### Study design

**Fig. 1:**
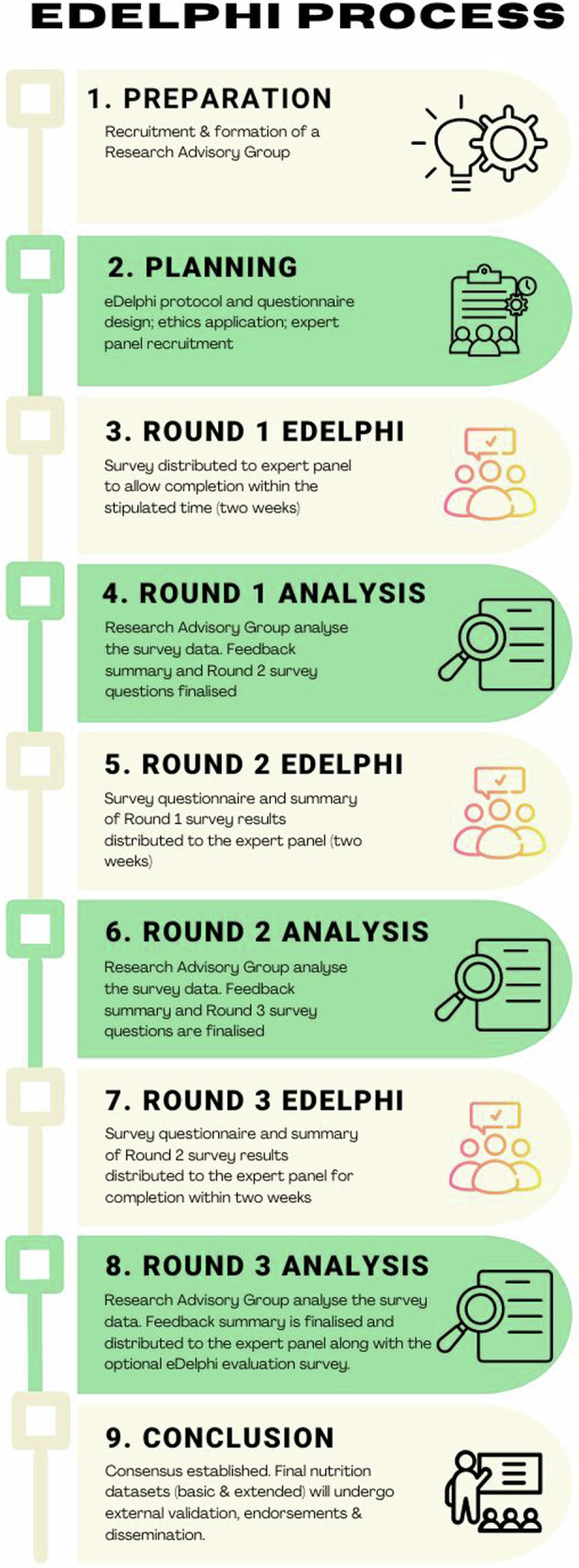
Flow diagram of the process used for eDelphi approach.

A three-round eDelphi (Fig. 1) [17] guided by Conducting and REporting DElphi Studies (CREDES) guidelines [18], will be employed for this study. This method is selected as it aligns well with the objectives of the study and has been proven to be effective in reaching consensus on emerging and underexplored topics in healthcare research [19].

### Study oversight

A Research Advisory Group (RAG) was established by soliciting expressions of interest from the ISCoS Nutrition Special Interest Group. The representative group consists of a dietitian from Sydney, Australia (PI), with over two decades of clinical experience; a dietitian from London, United Kingdom (SW), with more than a decade of expertise; two academics specialising in SCI and nutrition research from Chicago, Illinois (SLL) and Miami, Florida (GJF); and a health psychologist and academic from London, United Kingdom (SPH), with extensive experience in applied health research. Due to the governance and oversight role in the study, RAG members will refrain from participating directly in the eDelphi surveys.

### Identification of the expert panel

An international group of experts with expertise in SCI, including academic researchers and clinical professionals from various backgrounds (e.g., dietetics, medicine, nursing, pharmacy, exercise physiology, and other allied health disciplines), will be recruited to participate in the eDelphi expert panel (EP). EP members will actively participate in the eDelphi surveys to rate statements and provide expert justification and feedback to enable RAG to assess responses to achieve consensus. The criteria that individuals must meet to qualify as EP members include (1) a minimum of three years of recent clinical and/or research experience in the field of SCI OR (2) published one or more papers in the field of SCI and/or nutrition in SCI, and (3) able to read and write in the English language.

### Recruitment and informed consent

A combination of purposive and snowball sampling will be used to recruit participants for the eDelphi survey [20] as Phase A strategy. A recruitment flyer with links and a quick response (QR) code to an eligibility screener (Supplement 1), participant information, and a consent form will be shared by email via ISCoS interest groups. Additionally, the RAG will distribute the recruitment flyer through their respective professional networks to reach a wider and more diverse pool of potential experts. A gentle email reminder will be sent in a week after initial email circulation. Recipients of the flyer will be encouraged to forward the recruitment flyer to other experts, utilising a snowball strategy while maintaining the confidentiality and privacy of potential participants. If the initial recruitment strategies do not achieve the target sample size of 15 participants within two weeks of the initial email distribution, a secondary recruitment phase (Phase B) will be implemented during the International Nutrition Special Interest Group meeting. All eligible participants must provide informed consent as approved by the ethics committee at the host institution before participating in the survey. Those who consent will be prompted to complete a brief demographics survey (Supplement 2) to gather information on their professional role, age, sex, geographical location, number of publications and years of experience.

### Sample size of the expert panel

Although no precise recommendations exist for EP size, a sample size of approximately 10 to 18 diverse members are deemed adequate to build consensus [17, 21]. Considering the dropout rate of 20–30% in eDelphi studies [22], a minimum sample size of 15 is considered sufficient to establish a consensus [21]. Although no upper limit of participants has been established, the recruitment timeframe will be managed to ensure that the minimum number of participants is reached while preventing an excessively large group from posing challenges to manage within available resources. Diversity will be ensured by seeking representation from various disciplines and other demographic factors (e.g., sex, age etc) from across the globe.

### eDelphi survey development

Surveys will be managed using the Research Electronic Data Capture (REDCap) platform, a secure online software that enables seamless data management for research [23]. Utilising predefined items initially created by RAG member (SW) refined during the initial ISCoS Nutrition Dataset Subgroup meeting as a preliminary reference, another RAG member (PI) developed the initial survey statements. This was done by modifying the existing items and incorporating additional statements based on practical knowledge and experience in the field. The statements are grouped into sections guided by the Nutrition Care Process terminologies commonly used within dietetic practice [24]. The survey was further refined via numerous iterations and feedback from all members of the RAG before being finalised (Supplement 3). This version will be pilot tested by four experts (dietitians and researchers) independent of the RAG for content validity (measuring what it intends to measure) and readability (Supplement 4). The feedback and modification of the survey will be managed using an iterative process by the RAG, guided by simple analysis of content validity index scores to ascertain content validity and clarity.

### Consensus

An interquartile range (IQR) of ≤1 (strong consensus) and >1 but ≤2 (consensus) will be accepted as consensus being reached [25, 26]. Mean and standard deviation (SD) will be calculated if data are normally distributed to measure convergence and stability [27]. Agreement will be established if ≥80% of the responses to a statement fall within the 4 (agree) or 5 (strongly agree) range on the 5-point Likert scale. Conversely, disagreement will be noted if ≥80% of the responses to a statement fall within the 1 (strongly disagree) or 2 (disagree) range on the Likert scale [18]. The RAG will use an iterative process via virtual meetings to deliberate on statements with <80% agreement or disagreement to decide whether to retain, revise, or discard items based on their contextual importance. This iterative process will involve review of qualitative data, critical value of the stability of disagreement and open team discussions using anonymised results [18].The frequency and proportions of responses will be calculated for non-Likert scale questions/statements where experts choose between a basic or extended dataset. Consensus will be established if a choice receives a response rate of ≥75%. Responses <75% will be reviewed by the RAG and revisited in the next eDelphi round. If consensus has been reached on the dataset statement, only the type of dataset question will be presented in subsequent rounds.

### eDelphi rounds procedure

The experts who agree to participate in the eDelphi survey will receive an email containing a QR code and weblink for accessing the survey in each round, with two weeks allowed for completion per round [18]. Automated email reminders will be sent to non-responders on the seventh and tenth days of each eDelphi round. To achieve consensus on the recommended dataset, the EP will be asked to rate their level of agreement using a standard 5-point Likert scale (1 = strongly disagree to 5 = strongly agree) and categorise each item into the basic or extended dataset or neither dataset during the three rounds.

- Round 1: The EP will be asked to rate the initial set of statements regarding developing the nutrition dataset for SCI. Each statement will be accompanied by a textbox for respondents to provide any additional questions or comments or suggest any extra items for inclusion in the dataset. Responses will be exported into Excel (Microsoft 365, Microsoft Corporation, Redmond, WA) by an RAG member (PI) for quantitative data aggregation and compilation of comments. De-identified descriptive data and free text comments from Round 1 will be summarised and shared as feedback for Round 2. Statements meeting pre-defined criteria for consensus and agreement will be excluded from Round 2. Statements not meeting these criteria but deemed critical by the RAG or supported by experts’ feedback will be reviewed iteratively for potential inclusion in Round 2. Based on these considerations, the RAG will then design the survey for the second eDelphi round.
- Round 2: The Round 2 weblink, QR code, and summarised feedback of aggregated Round 1 results and comments will be emailed individually to the EP. eDelphi panel members will be asked to rate the statements following the same steps as in Round 1. Additionally, new statements or suggestions from Round 1 deemed relevant by the RAG will be incorporated into this round for consideration. A summary report will be prepared highlighting changes and any emerging consensus. New items suggested by the EP and statements that failed to reach an agreement, or consensus will be included for a rating in Round 3.
- Round 3: The third eDelphi round will proceed to gather feedback and achieve consensus among the EP on the key components of the nutrition dataset for SCI. This round will serve as the final round, and the EP will not have the option to propose new data items. Following a process like that of earlier rounds, the RAG will analyse the revised ratings and comments from Round 3 to finalise a consensus on the components of the nutrition dataset for SCI, encompassing basic and extended data items. The RAG will deliberate on the EP’s collective input, focusing on the dataset’s format, structure, and accessibility to ensure usability by all stakeholders. A summary report will be compiled and emailed to the EP for feedback, along with a unique link for an optional post-participation evaluation survey to assess their eDelphi experiences and improvement suggestions.

### Data management and analysis

Quantitative data will be calculated using SPSS (IBM, Armonk, NY) and reported using descriptive statistics ascertaining the normality of data. Free-text comments will be managed using NVivo 14 (Lumivero (2023) Version 14, www.lumivero.com) and analysed qualitatively using a simple content analysis approach [28]. Emerging themes at each round will be used to assess stability, whereby no new themes generated at consecutive rounds will be considered as achieving stability [29].

### External validation of the dataset

The final basic and extended nutrition datasets from the eDelphi consensus will undergo an established, standard ISCoS approval process [30]. The ISCoS Nutrition SIG’s dataset working group will undertake the first review before this is passed onto the ISCoS SCI Dataset Committee. Based on their feedback, the SIG dataset working group will further refine the dataset. Then, the dataset will be submitted to the American Spinal Injury Association (ASIA) Board of Directors and ISCoS Scientific and Executive Committee for review and feedback. Based on their input, subsequent updates will be made upon ISCoS Nutrition SIG deliberation. The updated dataset will be circulated to relevant international organisations for input. Simultaneously, the dataset will be available on the ISCoS website for a month to seek wider input. After this, SIG dataset working group will further refine the dataset, which will incorporate collective feedback. The ASIA Board of Directors and ISCoS Scientific and Executive Committee will conduct a final review and approve the dataset. The approved dataset will then undergo review by the National Institutes of Health National Institute of Neurological Disorders and Stroke Common Data Elements (NIH NINDS CDE). The SIG dataset working group and RAG will then develop standardised variable names and a database structure for the basic and extended datasets. Identification of standard measurement approaches and tools followed by implementation and evaluation of the dataset will be considered as the next steps following the establishment of a consensus-based framework for nutrition datasets in SCI.

#### Ethics and dissemination

Ethics approval (2024/HE000939) was obtained from the University of Sydney Human Ethics Committee, and the protocol was registered with the Open Science Framework (https://osf.io/xdq9a). All relevant institutional guidelines and ethical requirements will be adhered to in the conduct of this study. As part of the ISCoS approval process, the finalised datasets will be published on the ISCoS and NIH NINDS CDE websites and in the official peer-reviewed journal of the ISCoS, Spinal Cord. Furthermore, the nutrition dataset will be disseminated through ISCoS website, academic publications, professional conferences (including the Annual Scientific Meetings of ISCoS and ASIA), and online social media platforms to promote its integration into clinical practice, clinical practice guidelines, educational resources, and research initiatives.

## Supplementary information

Supplementary Materials

## Data Availability

All data will be available within the published paper as part of the main article and Supplementary Materials.
