# Supplementary material for: Protocol for developing the nutrition dataset for the international spinal cord society: an international eDelphi approach
Source: Spinal Cord. 2025 Jul 4;63(8):432–6. doi: 10.1038/s41393-025-01102-z (PMC12316596; doi:10.1038/s41393-025-01102-z)
Supplement: Supplementary file 1 — Supplementary Materials [file 41393_2025_1102_MOESM1_ESM.pdf]

SUPPLEMENTARY MATERIALS

This document includes four supplementary materials, each clearly labelled to indicate its content.

Supplement 1: Eligibility Screener
Supplement 2: Short Demographics' Survey
Supplement 3: Round-1 Survey Questionnaire
Supplement 4: Excerpt from content validity form

Supplement 1: Eligibility Screener

Nutrition dataset in SCI eDelphi
Page 1

Eligibility Screener

Eligibility Screener, version 2, 18/5/24	____________________________
I can communicate in English language.	◯ Yes<br>◯ No
I possess a minimum of at least five years of recent experience in the field of spinal cord injury, (clinical practice and/or research roles).	◯ Yes<br>◯ No
I have published at least two papers in the field of spinal cord injury/nutrition in spinal cord injury as either a first or senior (last) author.	◯ Yes<br>◯ No

Supplement 2: Short Demographics' Survey

Confidential

Page 1

New Short Demographics Form

Please complete the survey below.

Thank you!

Please complete the survey below. Thank you!

1) Please specify your geographical location (e.g., Australia, United States of America etc.). ____________________

2) Please select the age group you belong to
- ○ 18-24 years
- ○ 25-34 years
- ○ 35-44 years
- ○ 45-54 years
- ○ 55-64 years
- ○ 65 years and over

3) Sex
- ○ Male
- ○ Female
- ○ Other
- ○ Choose not to answer

Professional role/occupation

	Dietitian	Speech Pathologist	Other allied health	Nurse	Medical Practictione r	Researcher	Other
4) Please select all that applies.	☐	☐	☐	☐	☐	☐	☐

5) If you have selected 'other' for the last question, please provide details here. If not, please write NA. ____________________

6) Please specify the years of professional experience in the field of SCI and/or nutrition in SCI ____________________

7) Please indicate the number of publications in the field. Please say "none" if no publications. ____________________

Supplement 3
Round 1 Survey Questionnaire

Study ID

____

Nutrition Dataset in Spinal Cord Injury - Introduction to eDelphi Approach and guidance for completing the Round-1 survey.

Dear expert panellist,

Welcome to Round-1 of the eDelphi process. Please ensure you read the introductory document before starting this survey. It has been sent to your email along with this survey link, or you can access it via the attachment provided here. Please retain this document for your reference as necessary.

GUIDANCE TO COMPLETE ROUND-1 SURVEY: This questionnaire contains statements rated using a 5-point Likert scale. Rate your response to each statement and utilise the text box following each one to explain your choice, if needed or offer additional comments. Please determine whether the data items belong to the basic dataset, the extended dataset (which already contains the basic data items), or neither if you think they are not suitable for inclusion in either dataset. Furthermore, feel free to provide feedback on modifying the data points or suggest any additional crucial data points.

Scoring Guidance: 1 = STRONGLY DISAGREE; 2 = DISAGREE; 3 = NEITHER; 4 = AGREE; 5 = STRONGLY AGREE

ROUND 1 SURVEY QUESTIONNAIRE Version 4, 10/10/24

SECTION 1: ANTHROPOMETRY

Body weight

	1	2	3	4	5
Please rate the importance of including body weight (measured or estimated)	○	○	○	○	○

Dataset type

	Basic	Extended only	Neither
Please select the dataset type that body weight should be part of	○	○	○

Please provide justification for your choice including any comments you may have here.

____

Height

	1	2	3	4	5
Please rate the importance of including height (measured or estimated)	○	○	○	○	○

Dataset Type

	Basic	Extended only	Neither
Please select the dataset type that height should be part of	◯	◯	◯

Please provide justification for your choice including any comments you may have here. ______________________________

Body Mass Index (BMI)

	1	2	3	4	5
Please rate the importance of including BMI (with measured/reported data)	◯	◯	◯	◯	◯

Dataset Type

	Basic	Extended only	Neither
Please select the dataset type that BMI should be part of	◯	◯	◯

Please provide justification for your choice including any comments you may have here. ______________________________

Waist circumference

	1	2	3	4	5
Please rate the importance of including waist circumference	◯	◯	◯	◯	◯

Dataset Type

	Basic	Extended only	Neither
Please select the dataset type that waist circumference should be part of	◯	◯	◯

Please provide justification for your choice including any comments you may have here. ______________________________

Skinfold thickness

	1	2	3	4	5
Please rate the importance of including skinfold thickness	◯	◯	◯	◯	◯

Basic	Extended only	Neither

Please select the dataset type that skinfold thickness should be part of	◯	◯	◯

Please provide justification for your choice including any comments you may have here. Include your recommendations for measurement tools and approaches (e.g., 4, 7, or 9 sites). __________________________________

Body Composition

	1	2	3	4	5
Please rate the importance of including body composition	◯	◯	◯	◯	◯

Body composition components

	Body fat	Lean mass	Segmental breakdown of the above	Fat free mass	Body water
Please choose all that needs to be included under body composition	☐	☐	☐	☐	☐

Dataset type

	Basic	Extended only	Neither
Please select the dataset type that body composition should be part of	◯	◯	◯

Please provide justification for your choice including any comments you may have here. Suggest measurement tools/approaches, unit of measurements, or other measures for consideration. __________________________________

SECTION 2: BIOCHEMISTRY

Kidney function tests: A set of tests including electrolytes, urea, creatinine, estimated glomerular filtration rate (eGFR), and others are tested to see how well the kidneys are functioning. They can be serum (derived post clotting with centrifugation), or plasma (obtained using anti-clotting agents) measures as guided by the pathology specifications.

	1	2	3	4	5
Please rate the importance of including sodium	◯	◯	◯	◯	◯
Please rate the importance of including potassium	◯	◯	◯	◯	◯

Please rate the importance of including chloride	○	○	○	○	○
Please rate the importance of including urea	○	○	○	○	○
Please rate the importance of including creatinine	○	○	○	○	○
Please rate the importance of including eGFR	○	○	○	○	○
Please rate the importance of including phosphorous	○	○	○	○	○
Please rate the importance of including calcium	○	○	○	○	○
Please rate the importance of including albumin	○	○	○	○	○
Please rate the importance of including glucose	○	○	○	○	○

Dataset Type

	Basic	Extended only	Neither
Please select the dataset type that sodium should be part of	○	○	○
Please select the dataset type that potassium should be part of	○	○	○
Please select the dataset type that chloride should be part of	○	○	○
Please select the dataset type that urea should be part of	○	○	○
Please select the dataset type that creatinine should be part of	○	○	○
Please select the dataset type that eGFR should be part of	○	○	○
Please select the dataset type that phosphorous should be part of	○	○	○
Please select the dataset type that calcium should be part of	○	○	○
Please select the dataset type that albumin should be part of	○	○	○
Please select the dataset type that glucose should be part of	○	○	○

Please provide justification for your choices including any comments you may have here. __________________________________

Liver function tests (LFTs): A set of tests which are done to check how well the liver is functioning.

	1	2	3	4	5
Please rate the importance of including Alanine aminotransferase (ALT)	○	○	○	○	○
Please rate the importance of including Alkaline phosphatase (ALP)	○	○	○	○	○
Please rate the importance of including Aspartate aminotransferase (AST)	○	○	○	○	○
Please rate the importance of including Gamma-glutamyl transferase (GGT)	○	○	○	○	○
Please rate the importance of including bilirubin	○	○	○	○	○
Please rate the importance of including total protein	○	○	○	○	○

Dataset Type

	Basic	Extended only	Neither
Please select the dataset type that ALT should be part of	○	○	○
Please select the dataset type that ALP should be part of	○	○	○
Please select the dataset type that AST should be part of	○	○	○
Please select the dataset type that GGT should be part of	○	○	○
Please select the dataset type that bilirubin should be part of	○	○	○
Please select the dataset type that total protein should be part of	○	○	○

Please provide justification for your choice including any comments you may have here.

____

Full blood Count (FBC): A set of tests done routinely to check general health.

	1	2	3	4	5
Please rate the importance of including White blood cell count (WBC)	○	○	○	○	○
Please rate the importance of including Red blood cell count (RBC)	○	○	○	○	○
Please rate the importance of including haemoglobin	○	○	○	○	○
Please rate the importance of including Mean cell volume (MCV)	○	○	○	○	○
Please rate the importance of including haematocrit	○	○	○	○	○
Please rate the importance of including platelet count	○	○	○	○	○

Dataset type

	Basic	Extended only	Neither
Please select the dataset type that WBC should be part of	○	○	○
Please select the dataset type that RBC should be part of	○	○	○
Please select the dataset type that haemoglobin should be part of	○	○	○
Please select the dataset type that MCV should be part of	○	○	○
Please select the dataset type that haematocrit should be part of	○	○	○
Please select the dataset type that platelet count should be part of	○	○	○

Please provide justification for your choice including any comments you may have here.

____

Trace elements: Minerals required in small amounts important for health and wound healing.

	1	2	3	4	5
Please rate the importance of including iron	○	○	○	○	○
Please rate the importance of including zinc	○	○	○	○	○

Please rate the importance of including copper	○	○	○	○	○
Please rate the importance of including selenium	○	○	○	○	○

Datset type

	Basic	Extended only	Neither
Please select the dataset type that iron should be part of	○	○	○
Please select the dataset type that zinc should be part of	○	○	○
Please select the dataset type that copper should be part of	○	○	○
Please select the dataset type that selenium should be part of	○	○	○

Please provide justification for your choice including any comments you may have here.

____

Iron studies: Set of tests to check iron levels in blood which may be indicated in response to relevant clinical signs or concerns of deficiency.

	1	2	3	4	5
Please rate the importance of including serum iron	○	○	○	○	○
Please rate the importance of including serum ferritin	○	○	○	○	○
Please rate the importance of including serum transferrin	○	○	○	○	○
Please rate the importance of including transferrin saturation	○	○	○	○	○
Please rate the importance of including total iron binding capacity (TIBC)	○	○	○	○	○

Dataset type

	Basic	Extended only	Neither
Please select the dataset type that iron should be part of	○	○	○
Please select the dataset type that ferritin should be part of	○	○	○
Please select the dataset type that transferrin should be part of	○	○	○

Please select the dataset type that transferrin saturation should be part of	○	○	○
Please select the dataset type that TIBC should be part of	○	○	○

Please provide justification for your choice including any comments you may have here.

____

Glycated Haemoglobin (HbA1c)

	1	2	3	4	5
Please rate the importance of including these other biochemical markers	○	○	○	○	○

Dataset Type

	Basic	Extended only	Neither
Please select the dataset type that HbA1c should be part of	○	○	○

Please provide justification for your choice including any comments you may have here.

____

Please suggest other biochemical parameters which are relevant indicating whether they are basic or extended dataset. It is important to note that several markers, such as lipid profiles, vitamin D, and C-reactive protein, are already incorporated in other datasets, thus they are excluded here to avoid overlap.

____

SECTION 3: CLINICAL AND PHYSICAL FINDINGS

Swallowing function: Functional swallow or Dysphagia (swallowing difficulty)

	1	2	3	4	5
Please rate the importance of including swallowing function	○	○	○	○	○

Dataset type

	Basic	Extended only	Neither
Please select the dataset type that swallowing function should be part of	○	○	○

Please provide justification for your choice including any comments you may have here.

____

Food allergies/intolerances

	1	2	3	4	5
Please rate the importance of including food allergies and intolerances	○	○	○	○	○

Dataset type

	Basic	Extended only	Neither
Please select the dataset type that food allergies and intolerances should be part of	○	○	○

Please provide justification for your choice including any comments you may have here.

____

Bowel function (neurogenic bowel presentation; colostomy/other)

	1	2	3	4	5
Please rate the importance of including bowel function	○	○	○	○	○

Dataset type

	Basic	Extended only	Neither
Please select the dataset type that bowel function should be part of	○	○	○

Please provide justification for your choice including any comments you may have here.

____

Mode of feeding (oral/enteral/parenteral/combination)

	1	2	3	4	5
Please rate the importance of including the mode of feeding	○	○	○	○	○

Dataset type

	Basic	Extended only	Neither
Please select the dataset type that mode of feeding should be part of	○	○	○

Please provide justification for your choice including any comments you may have here.

____

Malnutrition screening and assessment: Malnutrition refers to both spectrums, undernutrition and overnutrition which may be as a result of lack of or excessive intake of energy/nutrients due to multiple causes. In simple terms, undernutrition can manifest as loss of muscle and fat (body weight) and overweight as unintentional weight gain.

	1	2	3	4	5
Please rate the importance of including undernutrition screening	○	○	○	○	○
Please rate the importance of including undernutrition assessment	○	○	○	○	○
Please rate the importance of including overnutrition screening	○	○	○	○	○
Please rate the importance of including overnutrition assessment	○	○	○	○	○

Dataset type

	Basic	Extended only	Neither
Please select the dataset type that undernutrition screening should be part of	○	○	○
Please select the dataset type that undernutrition assessment should be part of	○	○	○
Please select the dataset type that overnutrition screening should be part of	○	○	○
Please select the dataset type that overnutrition assessment should be part of	○	○	○

Please provide justification for your choice including any comments you may have here.

____

Energy expenditure: Is the amount of energy used for body functions and activity. Measurements used include total daily energy expenditure, resting metabolic rate etc.

	1	2	3	4	5
Please rate the importance of including energy expenditure	○	○	○	○	○

Dataset type

	Basic	Extended only	Neither
Please select the dataset type that energy expenditure should be part of	○	○	○

Please provide justification for your choice including any comments you may have here. Please suggest measures and approaches determining the classification of each (basic/extended). E.g. Resting metabolic rate measured using Indirect Calorimeter etc. __________________________________

SECTION 4: DIET/FOOD AND NUTRITION HISTORY

Diet order/type: Therapeutic diets such as diabetes diet, low potassium diet etc (including cultural preferences) and texture-modified diets (soft diet; minced moist or smooth puree diet and thickened fluids etc).

	1	2	3	4	5
Please rate the importance of including therapeutic dietary requirements	○	○	○	○	○
Please rate the importance of including texture-modified dietary requirements	○	○	○	○	○

Dataset type

	Basic	Extended only	Neither
Please select the dataset type that therapeutic dietary requirements should be part of	○	○	○
Please select the dataset type that texture-modified dietary requirements should be part of	○	○	○

Please provide justification for your choice including any comments you may have here. __________________________________

Estimated nutritional intake (quantitative data): Macronutrients and micronutrients

	1	2	3	4	5
Please rate the importance of including estimated energy intake	○	○	○	○	○
Please rate the importance of including estimated protein intake	○	○	○	○	○

Please rate the importance of including other estimated macronutrients' intake	◯	◯	◯	◯	◯
Please rate the importance of including estimated micronutrients' intake	◯	◯	◯	◯	◯
Please rate the importance of including estimated saturated fat intake	◯	◯	◯	◯	◯
Please rate the importance of including estimated added sugar intake	◯	◯	◯	◯	◯
Please rate the importance of including alcohol intake	◯	◯	◯	◯	◯

Dataset type

	Basic	Extended only	Neither
Please select the dataset type that estimated energy intake should be part of	◯	◯	◯
Please select the dataset type that estimated protein intake should be part of	◯	◯	◯
Please select the dataset type that other estimated macronutrients' intake should be part of	◯	◯	◯
Please select the dataset type that estimated micronutrients' intake should be part of	◯	◯	◯
Please select the dataset type that estimated saturated fat intake should be part of	◯	◯	◯
Please select the dataset type that estimated added sugar intake should be part of	◯	◯	◯
Please select the dataset type that alcohol intake should be part of	◯	◯	◯

Please provide justification for your choice including any comments you may have here.

____

Estimated nutritional intake (qualitative data): Food groups (recommended core groups including vegetables, fruits, dairy, meat/alternatives and grains) and diet quality.

	1	2	3	4	5
Please rate the importance of including estimated intake of recommended core food groups	○	○	○	○	○
Please rate the importance of including diet quality assessment	○	○	○	○	○

Dataset type

	Basic	Extended only	Neither
Please select the dataset type that estimated intake of core food groups' should be part of	○	○	○
Please select the dataset type that diet quality should be part of	○	○	○

Please provide justification for your choice including any comments you may have here.

____

ORAL FLUID INTAKE

	1	2	3	4	5
Please rate the importance of including oral fluid intake	○	○	○	○	○

Dataset type

	Basic	Extended only	Neither
Please select the dataset type that oral fluid intake should be part of	○	○	○

Please provide justification for your choice including any comments you may have here.

____

ORAL NUTRITION SUPPLEMENTS

	1	2	3	4	5
Please rate the importance of including oral nutrition supplements	○	○	○	○	○

Dataset type

	Basic	Extended only	Neither
Please select the dataset type that oral nutrition supplements should be part of	○	○	○

Please provide justification for your choice including any comments you may have here. ______________________

ENTERAL/PARENTERAL NUTRITION SUPPLEMENTS

	1	2	3	4	5
Please rate the importance of including enteral/parenteral nutrition supplements	○	○	○	○	○

Dataset type

	Basic	Extended only	Neither
Please select the dataset type that enteral/parenteral nutrition supplements should be part of	○	○	○

Please provide justification for your choice including any comments you may have here. ______________________

Final Words as wrap up of Round 1 eDelphi survey

Please add any additional data you would like to include in the dataset indicating whether they should be categorised as basic or extended. ______________________

Please add any general comments you may have here. ______________________

Round 1 of the survey is now complete. We value your input. A summary of Round 1 survey feedback along with the Round 2 survey link will be sent to you in the next two weeks.

Thank you for your participation.

Supplement 4: Excerpt from content validity form

INSTRUCTIONS

The purpose of this rating tool is to assess the content validity of the Round-1 Questionnaire. The questionnaire has been designed by the advisory panel with vast experience and subject matter expertise in the field.

Please review each item and rate the following on the columns to the right:

1) The relevance of the item on a 4-point scale, where:
   - 1 = very irrelevant
   - 2 = irrelevant
   - 3 = relevant
   - 4 = very relevant

2) The clarity of each item on a 4-point scale, where:
   - 1 = very unclear
   - 2 = unclear
   - 3 = clear
   - 4 = very clear

Item	Relevance<br>*1=very irrelevant, 2=irrelevant, 3=relevant 4=very relevant*	Clarity<br>*1=very unclear, 2=unclear, 3=clear, 4=very clear*	Comments<br>*(on questions or response options)*
Section 1: Anthropometry			
**Body weight**<br>Please rate the importance of including body weight (measured or estimated)<br>☐ 1 ☐ 2 ☐ 3 ☐ 4 ☐ 5	☐ 1<br>☐ 2<br>☐ 3<br>☐ 4	☐ 1<br>☐ 2<br>☐ 3<br>☐ 4	
**Dataset type**<br>Please select the dataset type that body weight should be part of<br>☐ Basic ☐ Extended only ☐ Neither<br>Please provide justification for your choice including any comments you may have here. ____________	☐ 1<br>☐ 2<br>☐ 3<br>☐ 4	☐ 1<br>☐ 2<br>☐ 3<br>☐ 4	
**Height**<br>Please rate the importance of including height (measured or estimated)<br>☐ 1 ☐ 2 ☐ 3 ☐ 4 ☐ 5	☐ 1<br>☐ 2<br>☐ 3<br>☐ 4	☐ 1<br>☐ 2<br>☐ 3<br>☐ 4	
**Dataset type**<br>Please select the dataset type that height should be part of<br>☐ Basic ☐ Extended only ☐ Neither<br>Please provide justification for your choice including any comments you may have here. ____________	☐ 1<br>☐ 2<br>☐ 3<br>☐ 4	☐ 1<br>☐ 2<br>☐ 3<br>☐ 4	

Content validity form adapted from LaVela DoD (2024)
